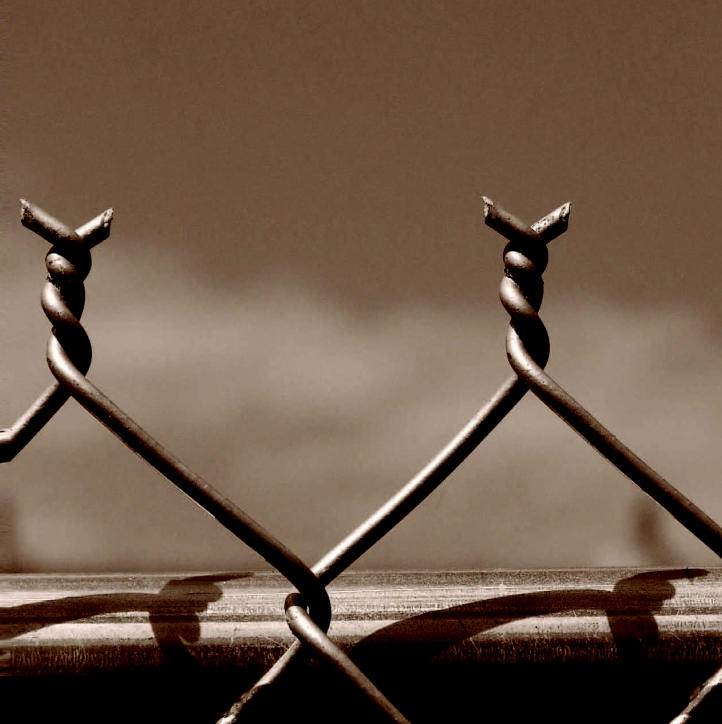# Obstructing Authority: Does the EPA Have the Power to Ensure Commercial Chemicals Are Safe?

**DOI:** 10.1289/ehp.114-a706

**Published:** 2006-12

**Authors:** Melissa Lee Phillips

Before 1976, the U.S. government had virtually no records of what chemicals were imported, manufactured, used, or released into the environment and no way of regulating these chemicals before they appeared on the market. That changed when Congress passed the Toxic Substances Control Act (TSCA), which required that chemical companies inform the EPA of the chemicals they currently used in American products and that they submit approval requests for any new chemical entering manufacture. Thirty years later, however, observers are asking how well TSCA has lived up to its initial promise and what powers the EPA actually has—questions Congress asked the U.S. Government Accountability Office (GAO) to investigate.

A report released in June 2005 by the GAO titled *Chemical Regulation: Options Exist to Improve EPA’s Ability to Assess Health Risks and Manage Its Chemical Review Program* claims that the TSCA legislation failed to empower the EPA to ensure the safety of chemicals used in the United States. On 2 August 2006, John B. Stephenson, director of natural resources and environment at the GAO, testified on the report’s findings before the Senate Committee on Environment and Public Works. Several other environmental scientists also testified in support of the GAO’s position that TSCA is in need of a major reworking if it is to adequately protect the health of U.S. citizens and the environment.

“Overhaul of TSCA is long overdue,” testified Lynn Goldman, former EPA assistant administrator for Prevention, Pesticides and Toxic Substances and now a professor of environmental health at the Johns Hopkins Bloomberg School of Public Health. “Minus congressional action on TSCA, we will continue to see the erosion of federal management of chemicals on many levels.”

Several other experts, however, testified that TSCA has mostly accomplished what it was designed to do. James B. Gulliford, the current EPA assistant administrator for Prevention, Pesticides and Toxic Substances, stated, “TSCA provides the agency with the necessary authority to ensure that new chemicals are adequately reviewed, that EPA can require reporting or development of information needed to assess existing chemicals, and that those chemicals that pose an unreasonable risk can be effectively controlled.”

## Life Under TSCA

When the EPA first began reviewing chemicals under TSCA in the late 1970s, about 62,000 chemicals were already in commerce, and TSCA did not require companies to provide any health or environmental safety data on these chemicals. Rather, Goldman says, “Existing chemicals were grandfathered in, and then EPA was given certain authorities to gather data to assess and to regulate them.”

In practice, regulating existing chemicals has been difficult, Stephenson says. The EPA has required testing of only about 200 of these grandfathered chemicals, and it has banned only five chemicals or chemical groups, according to Stephenson.

He says this is largely because TSCA places the burden of proof on the EPA to show that a chemical is dangerous rather than on the chemical company to show that it is safe. If EPA scientists suspect, based on existing information, that an existing chemical may pose a risk, the agency must go through a long rule-making process in order to get that information from the chemical industry, Stephenson says. This process can take years to complete.

EPA officials became discouraged with trying to regulate existing chemicals in the 1980s, Goldman says, when the U.S. Fifth Circuit Court of Appeals overturned the agency’s ban on asbestos. The court ruled that the EPA had failed to show two requirements of TSCA, Goldman says: that asbestos presented an “unreasonable risk” to public health and that banning it and replacing it with safer alternatives was the “least burdensome approach.” The court felt that controlling toxic substances after they are released into the environment is less burdensome than preventing pollution in the first place, Goldman told the Senate. Today numerous construction and automotive uses of asbestos are permitted.

Many EPA officials felt that it would be nearly impossible to prove that banning asbestos was in fact the least burdensome of all the possible alternatives. According to Goldman, by the time she began working with the EPA in 1993, the agency’s lawyers and the scientific and technical staff were very reluctant to try to regulate chemicals already in circulation. The asbestos ruling, she says, “was a kind of death knell for regulation of existing chemicals.”

## Gauging Potential

In the late 1990s, in an effort to supplement health and safety data on existing chemicals, the EPA introduced the High Production Volume (HPV) Challenge Program, under which chemical companies voluntarily provide test data on chemicals produced in volumes of 1 million pounds or more each year—some 2,800 chemicals (about 95% of the market by volume). Most chemical companies understand the benefit of participating in the HPV Challenge, says Kathleen Roberts, director of the Product Stewardship Team at the American Chemistry Council, the trade association representing U.S. chemical companies.

Providing safety data voluntarily is much faster than the traditional rule-making procedure, Roberts says. “It’s just much more efficient for the chemical companies to do it voluntarily,” she says, “and quite frankly, it’s much more efficient for EPA to accept that information.”

Voluntary negotiations are undoubtedly the fastest and easiest way to make sure that a chemical is safe, Goldman agrees, but industry also knows that EPA is “quite gun-shy” about using TSCA to regulate existing chemical uses. “When you don’t have the ability to regulate in your armamentarium, you are in a very weak negotiating position,” she says.

When a chemical company wishes to manufacture or import a new chemical, the company submits a premanufacture notice to the EPA 90 days before beginning production. This notice includes the chemical’s structure, information about processing and likely applications, estimated production volumes, and any testing data the company possesses, says Roberts. TSCA does not require companies to conduct specific testing on the health effects of new chemicals, however, so if the company hasn’t done any testing, the EPA uses computer modeling and its own professional judgment to compare the new chemical with other chemicals with similar structures, Roberts says.

Although modeling is a useful tool, it doesn’t always reveal potential dangers, Stephenson says. “The models are not always accurate in predicting physical chemical properties,” he said in his Senate testimony, “and the evaluation of general health effects is contingent on the availability of information on chemicals with similar molecular structures.” According to Roberts, however, “If there are no chemicals out there that EPA feels comfortable are similar enough, they’ll go back to that company and say, ‘You have to do this test for us.’ The company either does that test, or they withdraw their new chemical.” She says it’s a misperception to think that the EPA gives companies a free pass just because no routine testing is required for all chemicals. Goldman also indicates that the EPA’s examination of new chemicals is more thorough than that for those already on the market.

## Negotiating Changes

According to Gulliford, TSCA as it stands provides the EPA with the necessary tools “to ensure that chemicals are manufactured and used safely, and that the public and the environment are adequately protected.” Nevertheless, EPA press officer Enesta Jones says, “EPA is reviewing the recommendations by GAO to consider appropriate next steps.”

Many of the changes recommended by the GAO are simple word changes, Stephenson says, such as requiring that the EPA show a chemical poses a “significant” rather than “unreasonable” risk, or that it “may” rather than “will” present health risks. “It sounds small,” Stephenson says, “but, in practice, it makes a huge difference.”

Other recommendations include providing explicit authority for the EPA to enter into enforceable consent agreements under which chemical companies are required to conduct testing; giving the EPA the authority to require chemical substance manufacturers and processors to develop test data based on substantial production volume and the necessity for testing; and authorizing the EPA to share confidential industry information with states and foreign governments.

The American Chemistry Council’s stance is that TSCA’s framework is strong, and the EPA has the necessary authority to do what needs to be done, “[although] we can recognize that there may be some weaknesses associated with implementation of the tools,” Roberts says. For example, the EPA should make sure that they receive health data on the few remaining high-production chemicals that companies have not yet reported on under the HPV Challenge Program, she suggests. She adds, “I think EPA has had quite a bit of financial and staff resources that have been cut over the years, and so it’s been much more difficult for them to implement the law as envisioned by Congress.”

But she also notes that weaknesses in implementation do not mean that TSCA itself needs to be revised. “I think what we need to focus on is how to implement the tools better,” she explains. “Before we decide that the tools are no good, let’s see if there’s a way that we can use them better than we have in the past.”

Adjusting TSCA implementation will help make the statute more effective, Stephenson agrees, but it’s not the whole answer. “We think EPA can do some things without new legislation,” he says. “In other cases, there needs to be some amendments to . . . the act itself that Congress would have to undertake.”

TSCA legislation should be altered, Goldman says, but EPA scientists and administrators first need to gather more input from academic and industry scientists, environmental activists, lawyers, and other experts before Congress makes any changes. At present, she says, the situation is too polarized to expect that there can be progress toward reform. (Much of this polarization has arisen from widely differing reactions among industry, government, and activists to the stringent Registration, Evaluation and Authorisation of Chemicals regulatory framework proposed by the European Commission. Final adoption of this framework is expected by the end of 2006.)

According to Stephenson, TSCA could also include a risk-based framework, so that chemicals from families thought to be toxic would require more proof of safety than other chemicals. “We have to be sensitive to the industry’s point of view, as well; for EPA to require such tests on a whim would be a very expensive proposition,” he says. “It’s just that it’s so difficult for EPA to get something from the industry if it thinks it’s necessary. We think that burden can be shifted a little more towards the industry.”

## Figures and Tables

**Figure:**